# The Influence of a Place-Based Foundation and a Public University in Growing a Rural Health Workforce

**DOI:** 10.1007/s10900-018-0585-y

**Published:** 2018-10-20

**Authors:** Robert Reid, Evelyn Rising, Arthur Kaufman, Amanda Bassett, Martha Cole McGrew, Helene Silverblatt, Michael Haederle

**Affiliations:** 1 JF Maddox Foundation, Hobbs, NM USA; 2 Office for Community Health, University of New Mexico, Albuquerque, USA; 3 Family & Community Medicine, University of New Mexico Health Sciences Center, The University of New Mexico, MSC09 5065, 1 University of New Mexico, Albuquerque, NM 87131 USA; 4 University of New Mexico School of Medicine, Albuquerque, USA; 5 Family & Community Medicine, University of New Mexico Health Sciences Center, Albuquerque, USA; 6 University of New Mexico Health Sciences Center, Albuquerque, USA

**Keywords:** Private foundations, Rural health workforce, Public–private partnership, Community heath

## Abstract

An unlikely partnership between a private, place-based foundation and the University of New Mexico’s Office for Community Health resulted in an innovative approach for addressing a critical shortage of health professionals in an isolated, rural setting in the southeastern corner of New Mexico. Many place-based private foundations are focused locally and are naturally disinclined to engage distally located public universities for local projects. Large public universities do not often focus resources on small communities located far from their campuses. However, this unusual partnership resulted in a compelling vision of how atypical partners can collaborate in a way that is uniquely beneficial for a rural setting. Combining the entrepreneurial nature, flexible discretionary grant-making and local convening capabilities of a private foundation with the comprehensive set of resources of a public university allowed for a genuinely community-based approach in overcoming longstanding and systemically acute shortages in the local health care delivery workforce. Multi-party agreements were developed involving the JF Maddox Foundation, a local community college, local community hospitals and the University (the state’s only academic health center, based in Albuquerque), to engage both the University and local partners in ways that allowed for an entirely new approach to more effectively recruit, support, and retain local health care professionals. Results included significant increases in recruitment of key health care professionals, a more cohesive medical community, a school-based clinic and support for other community challenges, including prevention of teen pregnancy. The University has since exported this model to other rural communities in the state.

## Background, Community Health

Perhaps no other place in New Mexico captures the extreme isolation and impossible vastness of the state’s rural areas as dramatically as Lea County and its largest municipality, Hobbs. Isolated on the open plains in New Mexico’s far southeastern corner, Hobbs is just four miles from the Texas border—90 miles from Midland/Odessa, Texas, and a 5-h drive from Albuquerque, N.M.

The area was homesteaded in 1871 by ranchers. The natural terrain and conditions were so rough that many of the original structures were built partially underground for protection against the elements. The small town of Hobbs emerged in 1907 and blossomed into a busy municipality after oil was discovered in 1926 [1]. The community has been perpetually challenged by the highly cyclical economic trends of the oil and gas industry. This has resulted in waves of economic expansion and contraction tied to the boom-bust cycles of the energy business. Lea County was created in 1917, with a land mass three times the size of Rhode Island [1].

Today, Hobbs is one of the fastest-growing small cities in the country. The population is estimated at approximately 47,000 (2016 UNM Bureau of Business and Economic Research estimate, CCD), with an additional 75,000 people in the surrounding area [2]. The population is expanding, thanks to vast oil reserves in the Permian Basin recently unlocked by fracking and new horizontal drilling technologies. Hobbs is a diverse community, estimated at 65% Hispanic (based upon local school enrollment), many of whom are recent immigrants, and 8% African American (the highest percentage of any city in the state).

Like many rural communities, Hobbs faces persistent social problems, including high rates of teen pregnancy, substance abuse, asthma and many other health risks, as well as housing insecurity. It suffers from a lack of high-wage jobs, resulting in significant economic disparities. Median household incomes range from a low of $26,087 to a high of $100,455 (City-data.com—2016 updated census). Extreme differences in median household income are associated with location and are compounded by adverse social determinants of health and limited access to local medical care.

Hobbs is under-represented in terms of economic equity in the New Mexico Legislature, due to its sparse population and distance from the state capital, Santa Fe. While communities throughout Lea County contribute significantly to the state’s tax revenue, they rarely receive significant state support. For example, for many years the Hobbs Municipal School District averaged 83rd (out of 89 school districts) in state education funding per pupil [3].

But Hobbs has a strong entrepreneurial spirit and sense of independence (it often refers to itself as Hobbs, America, rather than Hobbs, New Mexico). Lea County residents share a pioneering spirit, and the vast majority of community assets in Hobbs are home-grown and self-funded. The state’s largest private foundation, the JF Maddox Foundation, came into existence in a similar manner.

The Foundation was founded by local entrepreneur Jack Maddox and his wife, Mabel, with very modest resources. It has been carefully managed and developed into a very significant philanthropic enterprise. Over its life, the Foundation has distributed approximately $150 million for charitable purposes, with a remaining endowment of approximately $260 million. The Foundation focuses almost exclusively on improving the quality of life for people living in communities of Lea County. This has resulted in significant programs of work in community/economic development, pre-K, K-12, higher education, social services, arts and culture, and recreation/quality of life. While the Foundation has robust programs of work in many areas, it had made little prior investment in the health sector.

## Limited Access to Health Care

Inadequate access to health care poses a significant challenge in Lea County. A 2013 community health needs assessment made several troubling discoveries in this regard [4]. The study found a significant lack of access to health care in general, and primary care in particular, a problem cited as a community challenge that has persisted for decades. For this reason, many residents cross into Texas to seek even the most basic health care, resulting in great economic loss to this area of New Mexico.

The county’s two hospitals, in aggregate, retain fewer than half of Lea County patients who require hospitalization. This medical “out-migration” (estimated at more than 55 percent of patients) is more than twice the regional average and results in more than $140 million per year in lost institutional revenue, which translates into a substantial number of health-related jobs [4]. The study estimated that Hobbs has a deficiency of approximately 29 additional primary care practitioners and an equal number of specialists to meet existing demand. Recruitment and retention challenges in rural communities suggested it was unlikely Hobbs could overcome such significant deficiencies in health care practitioners.

Based upon a pre-existing relationship between a physician member of the board of the Foundation and the Vice Chancellor for Community Health at the University of New Mexico Health Sciences Center (AK), the Foundation’s CEO reached out to request assistance in addressing serious deficiencies in Lea County health care delivery. Private foundations are often highly relational, electing to work with people and organizations in which there is high trust and mutual commitment [7]. Their capacity for independent decision-making and highly flexible approach to grant-making permit unusual efficiency in providing essential charitable support [7].

This request came soon after the Health Sciences Center leadership implemented a new Vision statement, which read, “Working with community partners, UNM will help New Mexico make more progress in health and health equity than any other state by 2020” [5]. A positive response to the Foundation’s request aligned with this new vision statement.

The University and the Foundation initially focused on ways to increase rotations of medical students in Hobbs. Evidence suggests that prospects for effective recruitment and retention of health care practitioners are enhanced by early community engagement as students. Foundation personnel randomly interviewed students on the main campus in Albuquerque to better understand possible impediments that might affect rotation selection criteria from the perspective of students. Students reported that free and/or low-cost housing, food support and good clinical experiences were among the most desirable attributes in selecting among clinical rotation options. Hobbs’ remote location, combined with inelastic housing availability, made it relatively unattractive to students. It was decided that this needed to be addressed comprehensively before seeking to accelerate student rotations through Hobbs.

## Method

The Foundation provided access to important local community and health care stakeholders, acting as a convener of participants and a local partner for the University. The Foundation and University worked very closely together—at times, as one seamless entity [8]. A plan to develop Hobbs as an educational destination for University students and residents evolved over a year, with multiple visits, calls and draft plans. Multiple leadership changes at one of the local hospitals further delayed and modified the plan. What ultimately emerged were two three-way agreements: Agreement No. 1 was between the University, New Mexico Junior College (NMJC) and Lea Regional Medical Center, and Agreement No. 2 included the University, NMJC and the Foundation.

Agreement No. 1 set in place the protocols for NMJC to provide on-campus housing, Internet service and access to workout facilities close by the hospital, while Lea Regional Medical Center, and ultimately another local hospital (Nor-Lea), provided clinical experience and preceptors, and the University facilitated and supported student rotations to Hobbs. Agreement No. 2 resulted in a grant of approximately $340,000 from the Foundation to fund housing costs for University students at NMJC for a trial period of 12 years, and defined institutional relationships between the University, NMJC and the Foundation.

Thanks to the Foundation’s grant to NMJC, a suite of dorm rooms was designated for free use by University students and/or residents rotating in Hobbs for clinical rotations. Lea Regional Medical Center, which was within walking distance from NMJC (and where most of the students would receive their clinical training), offered the learners free meals. The University’s Office for Community Health committed to working with the Health Sciences Center’s School of Medicine and Colleges of Nursing and Pharmacy to promote Hobbs as an attractive learning site and facilitate student and resident clinical rotations there. The co-location of many different types of learners in both housing and rotations served to emphasize interdisciplinary educational principles as well.

An important element added to the program to increase its likelihood of success was the hiring of a local Health Extension Rural Officer (HERO) (ER), funded by University’s Office for Community Health. Adapting an agricultural cooperative extension model to health, New Mexico’s HERO program placed HEROs in different regions of the state, where their role is to link community health needs with University resources [6].

The Hobbs HERO’s role included helping orient University students to the community—its resources, community leaders, agencies and entertainment opportunities. The HERO was chosen from and by the community and played a key role maintaining important community–university links, trouble-shooting, finding areas for program improvement and broadening the University’s partnership with sectors of the community’s medical providers and others outside health. It is this element of intimate knowledge of the community, led by preceptors and HEROs who live in a given community, that allowed learners to see a community as “a place they could live,” rather than just an educational site. This was critical to more effectively recruiting to, and retaining providers in, a community. The University can place students in rural settings, but it depends upon the communities themselves to create the opportunities the HERO could present to students.

Each University college then created its own agreement with Lea Regional Medical Center to allow students to see patients. With the help of the HERO, local clinical preceptors were recruited and offered training in teaching methods. The program was well advertised locally, by word of mouth and in the press to create a welcoming atmosphere for learners. A second hospital, Nor-Lea, subsequently joined the initiative.

While the HERO was based in Hobbs, her work also extended 20 miles to the north to Lovington, where the second hospital was located. Finally, the HERO’s role as a broker for campus–community relationships went beyond student and resident clinical training into all academic mission areas—clinical services, research and policy, as well as education. This success was shared with Hobbs and with the UNM Health Sciences Center.

The program in Hobbs also aligned with the leadership at the UNM Health Sciences Center in developing a more trusted relationships with the local communities. This included what had been a highly fragmented community of health care providers. Health Sciences Center representatives were able to convene members of the local medical community to assess local needs and evaluate collective resources for responding. In addition to discussing local needs, strategies were formed for enhancing recruitment and retention strategies. This resulted in considerable expansion of local primary care capacity, as several participants successfully recruited and more effectively retained essential health care professionals. The University also expanded access to specialists through telemedicine system. Hobbs and Lea County became a testing site for these newer, community-engagement roles of the UNM HSC.

## Outcomes

The unlikely partnership between the University and the local Foundation significantly enhanced local health care capacity—improving both recruitment and retention. The University provided critical leadership, students and support for students and the community. The Foundation opened doors with key stakeholders and provided resources needed to accommodate the needs of University students while on rotation in Lea County. Once the relationship between the University and the Foundation developed momentum, other compartmentalized University initiatives began to consider Hobbs as a desirable site. This resulted in several outcomes:

- Recruiting a health workforce has been successful. In the past 3 years, the County’s two community hospitals have recruited from UNM programs five family physicians, two physician assistants, two dental hygienists and one emergency medical technician.
- Expanding specialist consult capacity by using University telemedicine technologies. The local hospital physicians now access a range of specialist consultations, from neonatologists to neurosurgeons.
- Reducing the County’s exceedingly high teen pregnancy rate. UNM provided research guidance in developing a new school-based clinic and trained local providers in insertion of Long-Acting Reversible Contraceptives.
- Reversal of a preconception among University students that a rotation in Lea County was undesirable. Lea County clinical rotations are now considered highly preferable.
- Encouraging collegiality and collaboration within the local medical community. The convening capability of the Foundation, combined with the politically neutral standing of the University, provided a platform for collaboration. A highly fractious medical community came together, through the facilitation of University leaders, and collaborated in new and unexpected ways.
- Disseminating the model created in Hobbs to other rural communities in New Mexico. The University–Foundation partnership resulted in a new understanding of how to extend valuable University resources to rural communities to address community-driven priorities.

## Lessons Learned

Many lessons were learned from this initiative:

- *The value of sustained collaboration* The most important and instructive part of the story is the sustained collaboration between the parties over the course of a decade. While the Foundation provided essential financial support for the initiative, it was also able to draw upon its convening abilities to bring multiple parties together in support. The University provided students and residents to the project, but its broader commitment was expressed by assigning a health extension coordinator, addressing teen pregnancy and mobilizing telemedicine technologies.
- *Value of long-term foundation investment* Typically, foundation funding averages 3 years. This would not have provided a sufficient runway to allow this program to become successful. Because the Foundation and its university partner committed to a long-term relationship, trust was built, allowing branching ideas to emerge and be acted upon, [7]
- *Unique value of private foundation* A place-based foundation with a long-term commitment to specific communities can provide a platform on which universities can extend value. The University’s partnership with the Foundation made possible a 12-year trial period with continual institutional commitment to the Initiative.
- *Value beyond funding* A private foundation’s capital is one-third financial, one-third intellectual, and one-third “convening authority.” [8] A place-based foundation with a professional staff willing to collaborate with an entity such as the University could support developing local strategies in overcoming challenges that might otherwise threaten a project.
- *Why the model is replicable* Diffusion of the model does not always require new money, but can emerge from individuals and programs working together locally in new and more productive ways—realizing that the value of their collaboration is more than the sum of its parts. Most states and regions share the same elements that exist in this model—a university, a community college, a community hospital and potential local funding source (examples include local foundations, non-profit hospitals required to demonstrate public benefit, and town, city and county governments eager to recruit a health workforce and grow the local economy).

## Conclusion

Public universities often fall short in reaching beyond their traditional roles of teaching students and conducting research. However, they often possess capabilities that can prove transformative to communities, even those located far away. Finding ways to unbundle such capabilities for distal benefit remains an area that deserves further study. However, this initiative demonstrated that a private foundation with a place-based focus can provide a platform on which local university involvement can become more efficient and effective. Neither the University nor the Foundation could likely have achieved the outcomes of this initiative unilaterally. The willingness to depart from convention while developing mutual trust and respect through a long-term partnership allowed both entities to combine their respective capabilities in ways that achieved remarkable benefits for the participating communities. This kind of collaboration proved effective at unbundling many valuable competencies of a major public university for local benefit in a rural setting far away from the campus.
